# Effect of prenatal corticosteroids on the maturation of renal tubules in preterm infants

**DOI:** 10.1590/2175-8239-JBN-2023-0035en

**Published:** 2024-11-22

**Authors:** Jenny Ponce, Javier Cieza, Reyner Loza, Cristian León, Claudia Nuñez-Mochizaki

**Affiliations:** 1 Hospital Nacional Docente Madre Niño San Bartolomé, Departamento de Pediatría, Unidad de Nefrología, Lima, Peru.; 2 Hospital Nacional Cayetano Heredia, Departamento de Medicina, Unidad de Nefrología, Lima, Peru.; 3 Hospital Nacional Cayetano Heredia, Departamento de Pediatría, Unidad de Nefrología, Lima, Peru.

**Keywords:** Preterm, Prenatal Steroids, Tubular Function, Beta 2-Microglobulin

## Abstract

**Introduction:**

The benefit of prenatal steroids in fetal lung maturation is well established, but their effect on the kidney has not been studied in detail in humans. Animal models have shown an increase in the expression of Na^+^/H^+^ exchangers, Na^+^-K^+^-ATPase pumps and aquaporin-1. This study aims to assess the effect of prenatal steroids on the maturation of renal tubules in preterms.

**Methods:**

21 preterms born between 24 and 34 weeks were included. Participants were divided into two groups according to their exposure to prenatal steroids. The maturation of tubular function was evaluated through the change of the fractional excretion of Na^+^, K^+^ and beta 2-microglobulin, the transtubular gradient of K^+^, urine pH, and osmolality.

**Results:**

The mean age was 30.57 ± 1.8 weeks and the mean weight was 1415 ± 267.8 grams. Baseline differences were not observed, except for a significant difference in the change in urinary excretion of beta 2-microglobulin (Group 1: 1 980 ± 9 075 ng/mL and Group 2: –7 274 ± 10 006 ng/mL, p = 0.04).

**Conclusions:**

Prenatal steroids showed no statistically significant difference on renal tubule maturation in preterms.

## Introduction

Every year, an estimated 15.22 million babies are born prematurely, which corresponds to approximately 10% of all births worldwide. In 2019, the number of deaths from premature births was registered at 0.66 million. More than 85% of deaths from neonatal preterm birth globally and approximately 90% in South Africa are among newborns aged 0 to 6 days^1^.

The mortality and morbidity of premature infants increases with the degree of immaturity^2^. Since the 1960s, corticosteroid therapy has been used for premature labor. This therapy resulted in a decrease in respiratory distress syndrome and mortality rate among premature babies less than 34 weeks gestation^3^. Several studies have evaluated the risks and benefits of this therapy. Experiments on animals have found a positive effect on kidney maturation^4,5,6,7,8^.

The preterm newborn is characterized by immaturity in maintaining acid-base and electrolyte homeostasis. During the first postnatal week, several processes involved in renal acidification and transport of ions undergo maturational changes that help the newborn to adjust to life outside the uterus. After birth, the progressive increase of hormones plays an important role in the initiation or acceleration of several developmental processes^9^.

Animal studies have described an increase in Na^+^/H^+^ exchanger activity (NHE) in proximal tubules after corticosteroid treatment by a direct effect on the stimulation of NHE3 gene transcription. An increase in exocytic insertion of NHE3 into the apical membrane from the intracellular pool has also been reported^4,6,10^. In addition, corticosteroids stimulate mRNA expression of ß-adrenergic receptors, which enhance the effect of catecholamines on the activity of NHE. This Na^+^/H^+^ exchanger is the main mediator of the proximal reabsorption of the filtered bicarbonate^11^. NHE activity and bicarbonate reabsorption are decreased in neonates. Postnatal increase of NHE improves the excretion of ammonia and acids allowing the kidney to adapt to acid overload^12,13^. Corticosteroids also have an effect on the maturation of the Na^+^-K^+^-ATPase, essential for establishing the electrochemical gradient in renal cells, transport of Na^+^ and excretion of K^+^ through the body^7^, and expression of aquaporin-1 and water transport in the kidney^8^.

In term infants, the decrease in fractional excretion of Na^+^ after 24 hours postpartum is a reflection of this postnatal kidney maturation. However, this maturation may be incomplete in preterms^7^. During the first week of life, urinary excretion of Na^+^ and the fraction excreted are high and inversely proportional to maturity^14^. With advancing gestational age and postnatal age, the conservation of Na^+^ becomes more efficient as a result of maturation. Several mechanisms have been proposed, including the effect of corticosteroids^14, 15^.

There are few and inconclusive studies in humans currently that evaluated the effect of steroids on the kidney. This study aims to assess the effect of prenatal corticosteroids on the maturation of renal tubules in preterm infants.

## Methods

A descriptive, analytical, independent group study was performed. All 24–34 week preterm infants born in a period of one year at Cayetano Heredia Hospital and San Bartolomé Hospital in Lima – Peru were included. Infants with perinatal asphyxia, kidney injury, renal or urinary tract malformations, or use of any diuretics during the first 7 days of life were excluded. Infants of mothers who received an incomplete course or more than one full course of prenatal steroids or whose last dose of steroids was administered more than 7 days before delivery were also excluded.

Patients were divided into two groups according to prenatal exposure to steroids. Preterm infants whose mothers were in imminent delivery or whom had a medical condition that prevented administration of steroids were included in the control group. Infants were included in the steroid group if their mother received one full course of 2 doses of betamethasone (12 mg within a 24-hour interval between doses) or 4 doses of dexamethasone (6 mg within an interval of 12 hours between doses). The decision to treat mothers with a course of steroids was determined entirely by the department of Gynecology and Obstetrics of the hospital according to its protocol.

Data were obtained from the patient’s medical records. Gestational age was determined by the date of last period or the first trimester ultrasound. Weight below the 10^th^ percentile according to Lubchenco’s growth curve was defined as small for gestational age. Renal function was evaluated after 24 hours of birth. A 50% increase of creatinine value compared with the mother’s creatinine or a value above 1.5 mg/dL with a normal maternal renal function was considered renal injury. Blood and urine samples were collected from all participants on the first and seventh postnatal days to assess the immediate effect of the prenatal exposure of steroids (1^st^ day of life) and its effect on postnatal maturation (measured by the delta of variables between the 7^th^ and 1^st^ days of life). Serum and urine creatinine (Cr), electrolytes, osmolality (Osm) and beta 2-microglobulin (B2M) were measured to calculate the following variables:

1.
$Fractionalexcretionofsodium:FeNa\%=\frac{{\left[{\text{Na}}^{+}\right]}_{\left(urine\right)}\times {\left[\text{Cr}\right]}_{\left(serum\right)}}{{\left[{\text{Na}}^{+}\right]}_{\left(serum\right)}\times {\left[\text{Cr}\right]}_{\left(urine\right)}}\times 100$

2.
$Fractionalexcretionofpotassium:FeK\%=\frac{{\left[K^{+}\right]}_{\left(urine\right)}\times {\left[\text{Cr}\right]}_{\left(serum\right)}}{{\left[K^{+}\right]}_{\left(serum\right)}\times {\left[\text{Cr}\right]}_{\left(urine\right)}}\times 100$

3.
$Fractionalexcretionofbeta2microglobulin:FeB2M\%=\frac{{\left[B2M\right]}_{\left(urine\right)}\times {\left[\text{Cr}\right]}_{\left(serum\right)}}{{\left[B2M\right]}_{\left(serum\right)}\times {\left[\text{Cr}\right]}_{\left(urine\right)}}\times 100$

4.
$Transtubularpotassiumgradient:TTKG=\frac{{\left[K^{+}\right]}_{\left(urine\right)}\times {\left[Osm\right]}_{\left(serum\right)}}{{\left[K^{+}\right]}_{\left(serum\right)}\times {\left[Osm\right]}_{\left(urine\right)}}\times 100$

Blood bicarbonate and blood and urine pH were also measured. The glomerular filtration rate (GFR) was calculated using the Schwartz formula^16^.

Spontaneously voided urine samples were collected using a urine bag attached to the genital area. For pH determination, the urine sample was placed in a container with thymol and processed within a maximum of 4 hours to prevent loss of CO_2_ and urine alkalinization. A pH processor (microprocessor-based Ben PH23 pH/mV/°C meter^®^) was used. The Jaffé method was used to measure creatinine levels and the chemo-luminescence method was used for beta 2-microglobulin. Serum and urinary electrolytes were measured by ion selective electrodes and osmolality was measured by an osmometer (The Advanced™ model 3D3 Osmometer). All samples were kept refrigerated until processing.

The size of the groups was calculated assuming a 25% difference in average values in the populations studied, 20% standard deviation, 95% confidence interval, and 80% study power. Statistical program Power and Sample Size Calculation version 1.0.17 was used. A sample size of 11 subjects in each group was estimated. For data analysis, all variables were registered in a database developed in the SPSS V.11 program. Each group’s variables were expressed as mean ± standard deviation. Student’s t test was used for comparison of means of independent groups. For qualitative variables the chi square test was used. P < 0.05 was accepted as a statistically significant difference. Finally, a multiple linear regression analysis was performed to explore whether any changes observed in the study variables were related to other variables that could influence the outcome. The final regression model with statistical significance was presented.

The study was approved by the ethics committee of Cayetano Heredia University and of the hospitals where the study was performed. An informed consent from a parent or legal guardian was obtained.

## Results

Twenty-one premature infants between 27 and 33 weeks of gestational age were included in the study (11 in the steroid group and 10 in non-steroid group). There were no significant differences in demographic characteristics (Table 1). All infants were hemodynamically stable and none received inotropic support, diuretic therapy, or blood transfusion during the first 7 days of life. Only two patients in the steroid group received insulin for hyperglycemia. For the first 24 hours, preterm infants were hydrated with electrolyte-free dextrose with a glucose infusion rate of 4–6 mg/kg/min adjusted to blood glucose levels. Electrolytes were recently added after 24 hours. 7 of the babies required parental nutrition (2 in the steroid group) and after the first seven days, 11 infants were entirely fed with breast milk or preterm infant formula (5 in the steroid group).

**Table 1.** Demographic data

Characteristic	Non-steroid group	Steroid group	*P* value
Patients	10	11	
Gestational age (weeks)	30.5 ± 1.8 (29–34)	30.6 ± 1.8 (27–34)	n.s.
Birth weight (g)	1461.5 ± 261.7 (985–1895)	1372.7 ± 278.7 (1000–1815)	n.s.
Length at birth (cm)	40.8 ± 2.4	38.7 ± 2.6	n.s.
Male Sex	5/10 (50%)	5/11 (45.5%)	n.s.
Small for gestational age	1/10 (10%)	1/11 (9%)	n.s.
Cause of prematurity			
Premature rupture of membranes	3/10 (30%)	6/11 (54.5%)	n.s.
Preeclampsia/eclampsia	3/10 (30%)	1/11 (9.1%)	
Third trimester bleeding	2/10 (20%)	0/11 (0%)	
Unknown	2/10 (20%)	4/11 (36.4%)	
Neonatal Sepsis	5/10 (50%)	8/11 (72.7%)	n.s.
Respiratory distress syndrome	7/10 (70%)	6/11 (54.5%)	n.s.
Phototherapy	9/10 (90%)	8/11 (72.87%)	n.s.

Note: Data are presented as mean ± standard deviation with minimum and maximum in parenthesis and numbers with percentages in parenthesis.

At immediate evaluation (first day of life), no significant difference was found between the two groups. No significant difference was found also in the variables measured 7 days after birth, except for the serum level of chlorine, which was greater in the group not exposed to prenatal steroids (Table 2).

**Table 2.** Outcomes at 1^st^ and 7^th^ day of life according to the study groups

	1^st^ day of life			7^th^ day of life		
	Non-steroid group (X ± SD)	Steroid group (X ± SD)	*P* value	Non-steroid group (X ± SD)	Steroid group (X ± SD)	*P* value
Serum creatinine (mg/dL)	0.7 ± 0.1	0.7 ± 0.1	n.s.	0.57 ± 0.07	0.60 ± 0.15	n.s.
GFR (ml/min)	18.7 ± 3.9	17.6 ± 2.8	n.s.	24.2 ± 5.4	21.4 ± 3.9	n.s.
Serum Na^+^ (mEq/L)	144.6 ± 5.1	141.8 ± 0.1	n.s.	138.3 ± 3.8	135.3 ± 5.6	n.s.
Serum K^+^ (mEq/L)	4.2 ± 0.7	4.5 ± 1.0	n.s.	4.6 ± 0.6	4.8 ± 0.7	n.s.
Serum Cl^–^ (mEq/L)	113.9 ± 3.4	112.2 ± 5.6	n.s.	111.6 ± 4.9	104.8 ± 5.7	**0.01**
Urinary Na^+^ (mEq/L)	62.9 ± 31.8	74.2 ± 37.2	n.s.	46.9 ± 24.6	62.3 ± 54.4	n.s.
Urinary K^+^ (mEq/L)	16.4 ± 9.3	21.4 ± 17.2	n.s.	31.2 ± 25.9	21.4 ± 8.2	n.s.
Urinary Cl^–^ (mEq/L)	44.2 ± 30.3	62.3 ± 30.6	n.s.	72.3 ± 38.6	75.2 ± 49.4	n.s.
Urinary creatinine (mg/dL)	11.5 ± 4.3	11.2 ± 6.2	n.s	15.2 ± 7.1	13.1 ± 4.8	n.s.
Bicarbonate (mEq/L)	17.8 ± 2.2	18.7 ± 1.0	n.s.	19.2 ± 2.9	19.5 ± 3.4	n.s.
Serum pH	7.32 ± 0.1	7.28 ± 0.1	n.s.	7.31 ± 0.1	7.33 ± 0.1	n.s.
Urinary pH	7.08 ± 0.4	6.54 ± 1.0	n.s.	5.9 ± 0.4	5.7 ± 0.6	n.s.
Serum B2-microglobulin (ng/mL)	2 880.0 ± 567.9	3 062.6 ± 715.8	n.s.	2 834.6 ± 409.1	2 585.7 ± 435.0	n.s.
Urinary B2-microglobulin (ng/mL)	5 419.0 ± 3 105.2	13 213.5 ± 11 648.1	n.s.	7 399.2 ± 8 709.0	5 939.6 ± 4 303.8	n.s.
Serum osmolality (Osm)	296.0 ± 21.6	297.3 ± 8.0	n.s.	287.8 ± 10.3	282.7 ± 7.7	n.s.
Urinary Osm (mOsm/kgH_2_O)	167.5 ± 70.8	251.4 ± 125.4	n.s.	234.6 ± 137.0	250.8 ± 108.8	n.s.
FENa%	3.1 ± 2.3	3.7 ± 1.5	n.s.	1.6 ± 1.3	2.1 ± 1.4	n.s.
FEK%	30.0 ± 26.4	28.6 ± 13.9	n.s.	27.6 ± 20.0	22.0 ± 10.7	n.s.
FEB2M%	14.0 ± 10.4	26.1 ± 16.8	n.s.	9.1 ± 7.9	10.7 ± 8.5	n.s.
GTTK	7.5 ± 4.1	5.6 ± 3.2	n.s.	9.1 ± 6.5	6.1 ± 3.4	n.s.

Note: Data are presented as mean ± standard deviation.

In order to examine whether prenatal exposure to corticosteroids had an effect on postnatal maturation of renal tubules, we analyzed the delta of the variables in relation to time (Table 3). The delta of the urinary excretion of beta 2-microglobulin was the only variable that changed significantly (p = 0.039). The delta of the fractional excretion of beta 2-microglobulin did not reach statistical significance (p = 0.059). There was no statistically significant model using multiple linear regression (Table 4). A negative correlation between excretion of urinary beta 2-microglobulin at birth (1^st^ day of life) and the change in urinary beta 2-microglobulin was found in the group using prenatal corticosteroids (r = –0.932, p = 0.000). This correlation was not observed in the group without steroids (r = –0.287, p = 0.42).

**Table 3.** Outcomes of the change of different variables with time after delivery according to the study groups

	Non-steroid group (X ± SD)	Steroid group (X ± SD)	P value
Δ Serum creatinine (mg/dL)	–0.14 ± 0.14	–0.13 ± 0.19	n.s.
Δ GFR (mL/min)	5.0 ± 5.90	3.45 ± 4.95	n.s.
Δ Urinary creatinine (mg/dL)	3.72 ± 8.40	1.97 ± 7.67	n.s.
Δ Urinary Na^+^ (mEq/L)	–16.0 ± 28.1	–11.9 ± 56.7	n.s.
Δ Urinary K^+^ (mEq/L)	14.8 ± 30.8	–0.1 ± 17.9	n.s.
Δ Urinary Cl^–^ (mEq/L)	28.1 ± 41.9	12.9 ± 55.7	n.s.
Δ Bicarbonate (mEq/L)	1.5 ± 3.8	0.8 ± 3.7	n.s.
Δ Serum pH	–0.02 ± 0.1	0.05 ± 0.1	n.s.
Δ Urinary pH	–1.2 ± 0.6	–0.9 ± 1.3	n.s.
Δ Serum B2M (ng/mL)	–45.4 ± 632.7	–476.8 ± 913.3	n.s.
Δ Urinary B2M (ng/mL)	1 980.0 ± 9 075.0	–7 274.0 ± 10 006.0	0.04
Δ Urinary Osmolality (mOsm/kg)	67.1 ± 152.4	–0.6 ± 115.2	n.s.
Δ FENa%	–1.5 ± 2.5	–1.6 ± 1.8	n.s.
Δ FEK%	–2.5 ± 40.1	–6.7 ± 17.2	n.s.
Δ FEB2M%	–4.9 ± 12.7	–15.4 ± 11.3	n.s.
Δ GTTK	1.5 ± 9.2	0.5 ± 2.4	n.s.

Note: Data are presented as mean ± standard deviation.

**Table 4.** Assessment of variables affecting change in urinary beta 2-microglobulin. Final model of multiple linear regression

	Non standardized coefficients B	Std. error	Standardized coefficients beta	t	*P* value
(Constant)	1 763.39	1 209.95		1.457	0.16
Δ Urinary creatinine^a^	933.87	127.09	0.703	7.348	0.00
Δ FEB2M%	696.53	77.80	0.856	8.953	0.00

Note: ^a^Dependent variable: Δ urinary beta 2-microglobulin.

## Discussion

We evaluated the maturation of renal tubular function of preterm infants exposed and not exposed to steroids. Animal studies have demonstrated positive effects of prenatal corticosteroids on the maturation of renal tubules, however this effect has not yet been reproduced in humans probably due to the complexity^4,5,6,7,8^.

This study showed no differences in urinary sodium excretion or fractional excretion between the two groups. Also, there were no differences in factors that may affect sodium excretion, such as the use of inotropic agents or diuretics. The immature tubular function of preterms produces a higher urinary Na^+^ loss and a greater fractional Na^+^ excretion rate, and with advancing postnatal age, the conservation of Na^+^ becomes more efficient as a result of maturation (14). In animal studies, the use of prenatal steroids increases the expression of transporters that enhance Na^+^ reabsorption at the proximal tubule^6,8,9,12^. Results in our study showed an increase in the fractional excretion of sodium in both groups (more than 1%) as expected due to functional immaturity, but no significant difference was found between groups or in the retention of Na^+^ (serum sodium). Plasmatic sodium was found to be decreased in both groups, a value that correlates with increased urinary loss of this electrolyte. We assume that steroids have no effect on maturation at this level.

Regarding potassium, steroids could have had an effect on the expression and maturation of the Na^+^-K^+^-ATPase of cell membranes, which could prevent the shift of K^+^ from the intracellular to the extracellular space, thereby decreasing serum K^+^ and the renal filtered load by the kidney. However, we did not find significant differences in serum K^+^ or urinary excretion of K^+^. Also no difference was found in the fractional excretion of potassium, indicating that steroids have no effect on renal tubule maturation. The factors that could affect the balance of K^+^ were examined. There were no differences in variables that could affect the intracellular-extracellular distribution of K^+^, such as use of insulin, acidosis, and serum osmolality, or in those that could cause decreased K^+^ renal excretion, such as decreased glomerular filtration rate or blood transfusion. On the other hand, our study showed no significant difference in the transtubular potassium gradient, demonstrating that steroids had no effect on the handling of K^+^ in the distal renal tubule, and hence on the aldosterone response at this level.

There were no significant differences in urinary pH, serum pH levels, or serum bicarbonate, indicating that steroids had no effect on tubular acidification. The use of glucocorticoids has been postulated to have an effect on postnatal maturation in tubular acidification. Baum and Quigley^5^ studied this effect in rabbits. They found that urinary pH and urinary bicarbonate were lower in rabbits receiving prenatal dexamethasone, with no differences in blood levels, which could be a secondary effect of the acceleration of the maturation of luminal transporters Na^+^/H^+^ and basolateral Na^+^/HCO3^5^. A limitation of our study is that we did not measure urinary bicarbonate, which could have helped us to better recognize the acid-base imbalance produced by reduced tubular mass and lack of tubular maturity, or maybe an improvement of acid homeostasis induced by steroids. While it is true that an appropriated GFR, tubular secretion and absorption, and maintenance of acid-base equilibrium are not achieved until 2–3 ½ years of age when kidneys reach their adult size, we could have compared acidification properties between groups ^17, 18^. Although we did not measure urinary bicarbonate, we measured serum bicarbonate and urinary pH. During the first day of life, both groups had a slight decrease in serum bicarbonate, which correlated with a more alkaline urinary pH in comparison with the urinary pH measurement on the 7^th^ day of life, indicating increased serum bicarbonate and acidic urine. Results support the fact that although we are born with nephrogenesis completed, we lack complete maturation of renal capacity, an ability that increases over the first years of life.

We also studied the effect of steroids on the improvement of urinary concentrating ability and we did not find significant differences in urine osmolality levels despite the increase in the expression of aquaporin-1 seen in animal studies^13^.

In order to evaluate the maturation of renal proximal tubules, we also measured excretion and fractional excretion of beta 2-microglobulin^14^. We found a decrease in serum beta 2-microglobulin after birth in the group that used prenatal steroids, which, although not statistically significant in our study, could be indirectly reflected by a postnatal significant decreases in urinary excretion of beta-2-microglobulin in the steroid group, by reducing the filtered load of this protein and not increasing its tubular reabsorption because no significant differences in its fractional excretion were seen. However, the p value of 0.06 in the fractional excretion of beta 2-microglobulin indicates a potential association that may be confirmed by a study with a larger sample or with a longer follow-up period, and this was another limitation of our study.

The effect of steroids on the reduction of the filtered load of beta 2-microglobulin in the nephron could be due to the anti-lymphoproliferative effect on mononuclear cells, which are the main sources of this protein^19^. For this reason, we also evaluated the factors that affect the increase of beta 2-microglobulin such as maternal infection, neonatal sepsis, acidosis, or decreased renal filtration^20^, and there were no significant differences. The decrease in urinary excretion of beta 2-microglobulin confirms that steroids have no effect on renal proximal tubule maturation in the uterus or postnatal, decreasing the inflammatory process. Our findings were similar to the findings of MacKintosh et al.^21^, who also found no significant difference in tubular reabsorption of beta 2-microglobulin in the group exposed to betamethasone.

In conclusion, prenatal corticosteroids showed no statistically significant difference on renal tubule maturation in preterm infants, despite the positive results obtained in animal studies.
