# Supplementary material for: Effect of prenatal corticosteroids on the maturation of renal tubules in preterm infants
Source: J Bras Nefrol. 2024 Nov 22;46(4):e20230035. doi: 10.1590/2175-8239-JBN-2023-0035en (PMC11593545; doi:10.1590/2175-8239-JBN-2023-0035en)
Supplement: Translation

# Translation: Efeito dos corticosteroides pré-natais na maturação dos túbulos renais em recém-nascidos prematuros

**DOI:** 10.1590/2175-8239-JBN-2023-0035pt

**Authors:** Jenny Ponce, Javier Cieza, Reyner Loza, Cristian León, Claudia Nuñez-Mochizaki

**Affiliations:** 1 Hospital Nacional Docente Madre Niño San Bartolomé, Departamento de Pediatría, Unidad de Nefrología, Lima, Peru.; 2 Hospital Nacional Cayetano Heredia, Departamento de Medicina, Unidad de Nefrología, Lima, Peru.; 3 Hospital Nacional Cayetano Heredia, Departamento de Pediatría, Unidad de Nefrología, Lima, Peru.

**Keywords:** Prematuro, Esteroides Pré-natais, Função Tubular, Beta 2-Microglobulina

## Abstract

**Introdução:**

O benefício dos esteroides pré-natais na maturação pulmonar fetal encontra-se bem estabelecido, mas seu efeito sobre os rins ainda não foi estudado detalhadamente em humanos. Modelos animais demonstraram um aumento na expressão de trocadores Na^+^/H^+^, bombas de Na^+^-K^+^-ATPase e aquaporina-1. O objetivo deste estudo é avaliar o efeito dos esteroides pré-natais na maturação dos túbulos renais em prematuros.

**Métodos:**

Foram incluídos 21 prematuros nascidos entre 24 e 34 semanas. Os participantes foram divididos em dois grupos de acordo com sua exposição aos esteroides pré-natais. A maturação da função tubular foi avaliada por meio da alteração da fração de excreção de Na^+^, K^+^ e beta 2-microglobulina, do gradiente transtubular de K^+^, do pH urinário e da osmolalidade.

**Resultados:**

A média de idade foi de 30,57 ± 1,8 semanas e o peso médio foi de 1415 ± 267,8 gramas. Não foram observadas diferenças basais, exceto por uma diferença significativa na alteração da excreção urinária de beta 2-microglobulina (Grupo 1: 1 980 ± 9 075 ng/mL e Grupo 2: –7 274 ± 10 006 ng/mL; p = 0,04).

**Conclusões:**

Os esteroides pré-natais não demonstraram diferença estatisticamente significativa na maturação dos túbulos renais em prematuros.

## Introdução

Todos os anos, estima-se que 15,22 milhões de bebês nascem prematuramente, o que corresponde a aproximadamente 10% de todos os nascimentos em todo o mundo. Em 2019, o número de óbitos decorrentes de nascimentos prematuros foi registrado em 0,66 milhão. Mais de 85% dos óbitos decorrentes de partos pré-termo registrados mundialmente, e aproximadamente 90% na África do Sul, ocorrem entre recém-nascidos com 0 a 6 dias de idade^1^.

A mortalidade e a morbidade de recém-nascidos prematuros aumentam com o grau de imaturidade^2^. Desde a década de 1960, a terapia com corticosteroides tem sido utilizada para o trabalho de parto prematuro. Essa terapia resultou em uma redução da síndrome da angústia respiratória e da taxa de mortalidade entre bebês prematuros com menos de 34 semanas de gestação^3^. Diversos estudos avaliaram os riscos e benefícios dessa terapia. Experimentos em animais constataram um efeito positivo na maturação renal^4,5,6,7,8^.

O recém-nascido pré-termo é caracterizado pela imaturidade na manutenção da homeostase ácido-base e eletrolítica. Durante a primeira semana pós-natal, vários processos envolvidos na acidificação renal e no transporte de íons sofrem alterações maturacionais que ajudam o recém-nascido a se adaptar à vida extrauterina. Após o nascimento, o aumento progressivo de hormônios desempenha um papel importante no início ou na aceleração de diversos processos de desenvolvimento^9^.

Estudos em animais descreveram um aumento na atividade do trocador Na^+^/H^+^ (NHE) nos túbulos proximais após o tratamento com corticosteroides por meio de um efeito direto na estimulação da transcrição do gene NHE3. Também foi relatado um aumento na inserção exocítica de NHE3 na membrana apical a partir do *pool* intracelular^4,6,10^. Além disso, os corticosteroides estimulam a expressão de mRNA dos receptores ß-adrenérgicos, o que potencializa o efeito das catecolaminas na atividade do NHE. Esse trocador Na^+^/H^+^ é o principal mediador da reabsorção proximal do bicarbonato filtrado^11^. A atividade do NHE e a reabsorção de bicarbonato estão reduzidas em neonatos. O aumento pós-natal do NHE melhora a excreção de amônia e ácidos, permitindo que o rim se adapte à sobrecarga de ácidos^12,13^. Os corticosteroides também têm efeito na maturação da Na^+^-K^+^-ATPase, essencial para estabelecer o gradiente eletroquímico nas células renais, o transporte de Na^+^ e a excreção de K^+^ pelo corpo^7^, além da expressão da aquaporina-1 e do transporte de água nos rins^8^.

Em bebês nascidos a termo, a diminuição da fração de excreção de Na^+^ após 24 horas pós-parto é um reflexo dessa maturação renal pós-natal. No entanto, essa maturação pode ser incompleta em prematuros^7^. Durante a primeira semana de vida, a excreção urinária de Na^+^ e a fração excretada são elevadas e inversamente proporcionais à maturidade^14^. Com o avanço da idade gestacional e da idade pós-natal, a conservação de Na^+^ torna-se mais eficiente em decorrência da maturação. Diversos mecanismos foram propostos, incluindo o efeito dos corticosteroides^14, 15^.

Atualmente, existem poucos e inconclusivos estudos em humanos que avaliaram o efeito dos esteroides nos rins. O objetivo deste estudo é avaliar o efeito dos corticosteroides pré-natais na maturação dos túbulos renais em recém-nascidos prematuros.

## Métodos

Foi realizado um estudo descritivo, analítico e de grupos independentes. Foram incluídos todos os bebês prematuros de 24 a 34 semanas nascidos no período de um ano no Hospital Cayetano Heredia e no Hospital San Bartolomé em Lima, Peru. Excluímos os recém-nascidos com asfixia perinatal, injúria renal, malformações renais ou do trato urinário ou que tenham utilizado qualquer diurético durante os primeiros 7 dias de vida. Também foram excluídos os recém-nascidos cujas mães receberam um curso incompleto ou mais de um curso completo de esteroides pré-natais, ou cuja última dose de esteroides foi administrada mais de 7 dias antes do parto.

Os pacientes foram divididos em dois grupos de acordo com a exposição pré-natal a esteroides. Os recém-nascidos prematuros cujas mães estavam na iminência do parto ou que apresentaram uma condição médica que impedisse a administração de esteroides foram incluídos no grupo controle. Os bebês foram incluídos no grupo de esteroides caso suas mães tivessem recebido um curso completo de 2 doses de betametasona (12 mg em um intervalo de 24 horas entre as doses) ou 4 doses de dexametasona (6 mg em um intervalo de 12 horas entre as doses). A decisão de tratar as mães com um curso de esteroides foi determinada integralmente pelo departamento de Ginecologia e Obstetrícia do hospital, de acordo com seu protocolo.

Os dados foram obtidos dos prontuários médicos dos pacientes. A idade gestacional foi determinada pela data da última menstruação ou pela ultrassonografia do primeiro trimestre. O peso abaixo do percentil 10, de acordo com a curva de crescimento de Lubchenco, foi definido como pequeno para a idade gestacional. A função renal foi avaliada após 24 horas do nascimento. Um aumento de 50% no valor da creatinina em comparação com a creatinina da mãe ou um valor acima de 1,5 mg/dL com função renal materna normal foi considerado como lesão renal. Amostras de sangue e urina foram coletadas de todos os participantes no primeiro e no sétimo dia pós-natal para avaliar o efeito imediato da exposição pré-natal aos esteroides (1° dia de vida) e seu efeito na maturação pós-natal (medido pelo delta das variáveis entre o 7° e o 1° dia de vida). Creatinina (Cr) sérica e urinária, eletrólitos, osmolalidade (Osm) e beta 2-microglobulina (B2M) foram medidos para calcular as seguintes variáveis:

1.
$Fraçãodeexcreçãodesódio:FeNa\%=\frac{{\left[{\text{Na}}^{+}\right]}_{\left(urina\right)}\times {\left[\text{Cr}\right]}_{\left(plasma\right)}}{{\left[{\text{Na}}^{+}\right]}_{\left(plasma\right)}\times {\left[\text{Cr}\right]}_{\left(urina\right)}}\times 100$

2.
$Fraçãodeexcreçãodepotássio:FeK\%=\frac{{\left[K^{+}\right]}_{\left(urina\right)}\times {\left[\text{Cr}\right]}_{\left(plasma\right)}}{{\left[K^{+}\right]}_{\left(plasma\right)}\times {\left[\text{Cr}\right]}_{\left(urina\right)}}\times 100$

3.
$Fraçãodeexcreçãodebeta2microglobulina:FeB2M\%=\frac{{\left[B2M\right]}_{\left(urina\right)}\times {\left[\text{Cr}\right]}_{\left(plasma\right)}}{{\left[B2M\right]}_{\left(plasma\right)}\times {\left[\text{Cr}\right]}_{\left(urina\right)}}\times 100$

4.
$Gradientetranstubulardepotássio:TTKG=\frac{{\left[K^{+}\right]}_{\left(urina\right)}\times {\left[Osm\right]}_{\left(plasma\right)}}{{\left[K^{+}\right]}_{\left(plasma\right)}\times {\left[Osm\right]}_{\left(urina\right)}}\times 100$

O bicarbonato no sangue e o pH sanguíneo e urinário também foram medidos. A taxa de filtração glomerular (TFG) foi calculada usando a fórmula de Schwartz^16^.

Amostras por micção espontânea foram coletadas por meio de uma bolsa coletora presa à área genital. Para a determinação do pH, a amostra de urina foi colocada em um recipiente com timol e processada em um período máximo de 4 horas para evitar a perda de CO_2_ e a alcalinização da urina. Foi utilizado um processador de pH (medidor de pH/mV/°C baseado em microprocessador Ben PH23^®^). Os níveis de creatinina foram medidos pelo método de Jaffé e a beta 2-microglobulina pelo método de quimioluminescência. Os eletrólitos séricos e urinários foram medidos por eletrodos seletivos de íons e a osmolalidade foi determinada por um osmômetro (modelo *Advanced™ 3D3 Osmometer*). Todas as amostras foram mantidas refrigeradas até o processamento.

O tamanho dos grupos foi calculado com base em uma diferença de 25% nos valores médios das populações estudadas, desvio padrão de 20%, intervalo de confiança de 95% e poder do estudo de 80%. Utilizou-se o programa estatístico *Power and Sample Size Calculation* versão 1.0.17. Foi estimado um tamanho de amostra de 11 indivíduos em cada grupo. Para a análise dos dados, todas as variáveis foram registradas em um banco de dados desenvolvido no programa SPSS V.11. As variáveis de cada grupo foram expressas como média ± desvio padrão. O teste t de Student foi utilizado para comparação das médias de grupos independentes. O teste qui-quadrado foi usado para variáveis qualitativas. Um valor de P < 0,05 foi aceito como evidência de diferença estatisticamente significativa. Por fim, foi realizada uma análise de regressão linear múltipla para explorar se quaisquer alterações observadas nas variáveis do estudo estavam relacionadas a outras variáveis que podem influenciar o desfecho. Foi apresentado o modelo de regressão final com significância estatística.

O estudo foi aprovado pelo comitê de ética da Universidade Cayetano Heredia e dos hospitais onde o estudo foi realizado. Foi obtido o consentimento informado de um dos pais ou do responsável legal.

## Resultados

Vinte e um recém-nascidos prematuros entre 27 e 33 semanas de idade gestacional foram incluídos no estudo (11 no grupo de esteroides e 10 no grupo de não esteroides). Não houve diferenças significativas nas características demográficas (Tabela 1). Todos os bebês estavam hemodinamicamente estáveis e nenhum recebeu suporte inotrópico, terapia diurética ou transfusão sanguínea durante os primeiros sete dias de vida. Somente dois pacientes no grupo de esteroides receberam insulina para hiperglicemia. Durante as primeiras 24 horas, os recém-nascidos prematuros foram hidratados com dextrose sem eletrólitos, com uma taxa de infusão de glicose de 4 a 6 mg/kg/min ajustada aos níveis de glicose no sangue. Os eletrólitos foram adicionados imediatamente após 24 horas. Sete dos bebês necessitaram de nutrição parenteral (2 no grupo de esteroides) e, após os primeiros sete dias, 11 recém-nascidos foram exclusivamente alimentados com leite materno ou fórmula para bebês prematuros (5 no grupo de esteroides).

**Tabela 1.** Dados demográficos

Característica	Grupo não esteroide	Grupo de esteroides	Valor de *P*
Pacientes	10	11	
Idade gestacional (semanas)	30,5 ± 1,8 (29–34)	30,6 ± 1,8 (27–34)	n.s.
Peso ao nascer (g)	1461,5 ± 261,7 (985–1895)	1372,7 ± 278,7 (1000–1815)	n.s.
Comprimento ao nascer (cm)	40,8 ± 2,4	38,7 ± 2,6	n.s.
Sexo masculino	5/10 (50%)	5/11 (45,5%)	n.s.
Pequeno para a idade gestacional	1/10 (10%)	1/11 (9%)	n.s.
Causa da prematuridade			
Ruptura prematura das membranas	3/10 (30%)	6/11 (54,5%)	n.s.
Pré-eclâmpsia/eclâmpsia	3/10 (30%)	1/11 (9,1%)	
Sangramento do terceiro trimestre	2/10 (20%)	0/11 (0%)	
Desconhecida	2/10 (20%)	4/11 (36,4%)	
Sepse neonatal	5/10 (50%)	8/11 (72,7%)	n.s.
Síndrome da angústia respiratória	7/10 (70%)	6/11 (54,5%)	n.s.
Fototerapia	9/10 (90%)	8/11 (72.87%)	n.s.

Nota: Os dados são apresentados como média ± desvio padrão, com mínimo e máximo entre parênteses e números com porcentagens entre parênteses.

Na avaliação imediata (primeiro dia de vida), não foi encontrada diferença significativa entre os dois grupos. Nenhuma diferença significativa foi encontrada também nas variáveis medidas 7 dias após o nascimento, com exceção do nível sérico de cloro, que foi maior no grupo não exposto a esteroides pré-natais (Tabela 2).

**Tabela 2.** Desfechos no 1° e 7° dia de vida, segundo os grupos de estudo

	1° dia de vida			7° dia de vida		
	Grupo não esteroide (X ± DP)	Grupo de esteroides (X ± DP)	Valor de *P*	Grupo não esteroide (X ± DP)	Grupo de esteroides (X ± DP)	Valor de *P*
Creatinina sérica (mg/dL)	0,7 ± 0,1	0,7 ± 0,1	n.s.	0,57 ± 0,07	0,60 ± 0,15	n.s.
TFG (ml/min)	18,7 ± 3,9	17,6 ± 2,8	n.s.	24,2 ± 5,4	21,4 ± 3,9	n.s.
Na^+^ sérico (mEq/L)	144,6 ± 5,1	141,8 ± 0,1	n.s.	138,3 ± 3,8	135,3 ± 5,6	n.s.
K^+^ sérico (mEq/L)	4,2 ± 0,7	4,5 ± 1,0	n.s.	4,6 ± 0,6	4,8 ± 0,7	n.s.
Cl^–^ sérico (mEq/L)	113,9 ± 3,4	112,2 ± 5,6	n.s.	111,6 ± 4,9	104,8 ± 5,7	0,01
Na^+^ urinário (mEq/L)	62,9 ± 31,8	74,2 ± 37,2	n.s.	46,9 ± 24,6	62,3 ± 54,4	n.s.
K^+^ urinário (mEq/L)	16,4 ± 9,3	21,4 ± 17,2	n.s.	31,2 ± 25,9	21,4 ± 8,2	n.s.
Cl^–^ urinário (mEq/L)	44,2 ± 30,3	62,3 ± 30,6	n.s.	72,3 ± 38,6	75,2 ± 49,4	n.s.
Creatinina urinária (mg/dL)	11,5 ± 4,3	11,2 ± 6,2	n.s	15,2 ± 7,1	13,1 ± 4,8	n.s.
Bicarbonato (mEq/L)	17,8 ± 2,2	18,7 ± 1,0	n.s.	19,2 ± 2,9	19,5 ± 3,4	n.s.
pH sérico	7,32 ± 0,1	7,28 ± 0,1	n.s.	7,31 ± 0,1	7,33 ± 0,1	n.s.
pH urinário	7,08 ± 0,4	6,54 ± 1,0	n.s.	5,9 ± 0,4	5,7 ± 0,6	n.s.
B2-microglobulina sérica (ng/mL)	2 880,0 ± 567,9	3 062,6 ± 715,8	n.s.	2 834,6 ± 409,1	2 585,7 ± 435,0	n.s.
B2-microglobulina urinária (ng/mL)	5 419,0 ± 3 105,2	13 213,5 ± 11 648,1	n.s.	7 399,2 ± 8 709,0	5 939,6 ± 4 303,8	n.s.
Osmolalidade sérica (Osm)	296,0 ± 21,6	297,3 ± 8,0	n.s.	287,8 ± 10,3	282,7 ± 7,7	n.s.
Osm urinária (mOsm/kgH_2_O)	167,5 ± 70,8	251,4 ± 125,4	n.s.	234,6 ± 137,0	250,8 ± 108,8	n.s.
FENa%	3,1, ± 2,3	3,7 ± 1,5	n.s.	1,6 ± 1,3	2,1 ± 1,4	n.s.
FEK%	30,0 ± 26,4	28,6 ± 13,9	n.s.	27,6 ± 20,0	22,0 ± 10,7	n.s.
FEB2M%	14,0 ± 10,4	26,1 ± 16,8	n.s.	9,1 ± 7,9	10,7 ± 8,5	n.s.
GTTK	7,5 ± 4,1	5,6 ± 3,2	n.s.	9,1 ± 6,5	6,1 ± 3,4	n.s.

Nota: Os dados são apresentados como média ± desvio padrão.

Para examinar se a exposição pré-natal a corticosteroides teve efeito na maturação pós-natal dos túbulos renais, analisamos o delta das variáveis em relação ao tempo (Tabela 3). O delta da excreção urinária de beta 2-microglobulina foi a única variável que sofreu alteração significativa (p = 0,039). O delta da fração de excreção de beta 2-microglobulina não atingiu significância estatística (p = 0,059). Não houve nenhum modelo estatisticamente significativo utilizando regressão linear múltipla (Tabela 4). Foi encontrada uma correlação negativa entre a excreção de beta 2-microglobulina urinária ao nascimento (1° dia de vida) e a alteração na beta 2-microglobulina urinária no grupo que fez uso de corticosteroides pré-natais (r = –0,932; p = 0,000). Essa correlação não foi observada no grupo sem esteroides (r = –0,287; p = 0,42).

**Tabela 3.** Desfechos da mudança de diferentes variáveis ao longo do tempo após o parto, de acordo com os grupos de estudo

	Grupo não esteroide (X ± DP)	Grupo de esteroides (X ± DP)	Valor de P
Δ Creatinina sérica (mg/dL)	–0,14 ± 0,14	–0,13 ± 0,19	n.s.
Δ TFG (mL/min)	5,0 ± 5,90	3,45 ± 4,95	n.s.
Δ Creatinina urinária (mg/dL)	3,72 ± 8,40	1,97 ± 7,67	n.s.
Δ Na^+^ urinário (mEq/L)	–16,0 ± 28,1	–11,9 ± 56,7	n.s.
Δ K^+^ urinário (mEq/L)	14,8 ± 30,8	–0,1 ± 17,9	n.s.
Δ Cl^–^ urinário (mEq/L)	28,1 ± 41,9	12,9 ± 55,7	n.s.
Δ Bicarbonato (mEq/L)	1,5 ± 3,8	0,8 ± 3,7	n.s.
Δ pH sérico	–0,02 ± 0,1	0,05 ± 0,1	n.s.
Δ pH urinário	–1,2 ± 0,6	–0,9 ± 1,3	n.s.
Δ B2M sérica (ng/mL)	–45,4 ± 632,7	–476,8 ± 913,3	n.s.
Δ B2M urinária (ng/mL)	1 980,0 ± 9 075,0	–7 274,0 ± 10 006,0	**0,04**
Δ Osmolalidade urinária (mOsm/kg)	67,1 ± 152,4	–0,6 ± 115,2	n.s.
Δ FENa%	–1,5 ± 2,5	–1,6 ± 1,8	n.s.
Δ FEK%	–2,5 ± 40,1	–6,7 ± 17,2	n.s.
Δ FEB2M%	–4,9 ± 12,7	–15,4 ± 11,3	n.s.
Δ GTTK	1,5 ± 9,2	0,5 ± 2,4	n.s.

Nota: Os dados são apresentados como média ± desvio padrão.

**Tabela 4.** Avaliação das variáveis que afetam a alteração da beta 2-microglobulina urinária. Modelo final de regressão linear múltipla

	Coeficientes não padronizados B	Erro padrão	Coeficientes padronizados beta	t	Valor de *P*
(Constante)	1 763,39	1 209,95		1,457	**0,16**
Δ Creatinina urinária^a^	933,87	127,09	0,703	7,348	0,00
Δ FEB2M%	696,53	77,80	0,856	8,953	0,00

Nota: ^a^Variável dependente: Δ beta 2-microglobulina urinária.

## Discussão

Avaliamos a maturação da função tubular renal de recém-nascidos prematuros expostos e não expostos a esteroides. Estudos em animais demonstraram efeitos positivos dos corticosteroides pré-natais na maturação dos túbulos renais; no entanto, esse efeito ainda não foi reproduzido em humanos, provavelmente devido à complexidade^4,5,6,7,8^.

Esse estudo não demonstrou diferenças na excreção urinária de sódio ou na fração de excreção entre os dois grupos. Além disso, não houve diferenças nos fatores que podem afetar a excreção de sódio, como o uso de agentes inotrópicos ou diuréticos. A função tubular imatura de prematuros produz uma maior perda urinária de Na^+^ e uma taxa mais elevada de excreção fracionada de Na^+^. Com o avanço da idade pós-natal, a conservação de Na^+^ torna-se mais eficiente como resultado da maturação (14). Em estudos com animais, o uso de esteroides pré-natais aumenta a expressão de transportadores que melhoram a reabsorção de Na^+^ no túbulo proximal^6,8,9,12^. Os resultados do nosso estudo demonstraram um aumento na fração de excreção de sódio em ambos os grupos (mais de 1%), conforme esperado, devido à imaturidade funcional, mas não foi encontrada diferença significativa entre os grupos ou na retenção de Na^+^ (sódio sérico). O sódio plasmático mostrou-se diminuído em ambos os grupos, um valor que se correlaciona com o aumento da perda urinária desse eletrólito. Presumimos que os esteroides não têm efeito na maturação neste nível.

Em relação ao potássio, os esteroides podem ter exercido um efeito na expressão e maturação da Na^+^-K^+^-ATPase das membranas celulares, o que pode evitar o deslocamento de K^+^ do espaço intracelular para o extracelular, diminuindo assim o K^+^ sérico e a carga renal filtrada pelos rins. No entanto, não encontramos diferenças significativas no K^+^ sérico ou na excreção urinária de K^+^. Também não foi encontrada nenhuma diferença na fração de excreção de potássio, indicando que os esteroides não têm efeito sobre a maturação dos túbulos renais. Foram analisados os fatores que podem afetar o equilíbrio de K^+^. Não houve diferenças nas variáveis que podem afetar a distribuição intracelular-extracelular de K^+^, como uso de insulina, acidose e osmolalidade sérica, ou naquelas que podem causar diminuição da excreção renal de K^+^, como taxa de filtração glomerular reduzida ou transfusão sanguínea. Por outro lado, nosso estudo não mostrou diferença significativa no gradiente transtubular de potássio, demonstrando que os esteroides não tiveram efeito no manejo do K^+^ no túbulo renal distal e, portanto, na resposta à aldosterona nesse nível.

Não houve diferenças significativas no pH urinário, nos níveis séricos de pH ou no bicarbonato sérico, indicando que os esteroides não tiveram efeito na acidificação tubular. Postula-se que o uso de glicocorticoides tenha um efeito na maturação pós-natal da acidificação tubular. Baum e Quigley^5^ estudaram esse efeito em coelhos. Eles constataram que o pH urinário e o bicarbonato urinário eram mais baixos em coelhos que receberam dexametasona pré-natal, sem diferenças nos níveis sanguíneos, o que poderia ser um efeito secundário da aceleração da maturação dos transportadores luminais Na^+^/H^+^ e Na^+^/HCO3 basolateral^5^. Uma limitação do nosso estudo é que não medimos o bicarbonato urinário, o que poderia ter nos ajudado a reconhecer melhor o desequilíbrio ácido-base produzido pela redução da massa tubular e falta de maturidade tubular, ou talvez uma melhora da homeostase ácida induzida por esteroides. Embora seja verdade que uma TFG adequada, secreção e absorção tubular e manutenção do equilíbrio ácido-base não sejam alcançadas até os 2-3 ½ anos de idade, quando os rins atingem seu tamanho adulto, poderíamos ter comparado as propriedades de acidificação entre os grupos^17, 18^. Embora não tenhamos medido o bicarbonato urinário, mensuramos o bicarbonato sérico e o pH urinário. Durante o primeiro dia de vida, ambos os grupos apresentaram uma leve redução no bicarbonato sérico, que se correlacionou com um pH urinário mais alcalino em comparação com a medição do pH urinário no sétimo dia de vida, indicando aumento do bicarbonato sérico e urina ácida. Os resultados corroboram o fato de que, embora nasçamos com a nefrogênese concluída, falta-nos a maturação completa da capacidade renal, uma habilidade que aumenta ao longo dos primeiros anos de vida.

Também investigamos o efeito dos esteroides na melhora da capacidade de concentração urinária e não encontramos diferenças significativas nos níveis de osmolalidade urinária, apesar do aumento da expressão da aquaporina-1 observado em estudos com animais^13^.

Para avaliar a maturação dos túbulos proximais renais, também medimos a excreção e a fração de excreção de beta 2-microglobulina^14^. Constatamos uma redução na beta-2-microglobulina sérica após o nascimento no grupo que fez uso de esteroides pré-natais, o que, embora não tenha sido estatisticamente significativo em nosso estudo, pode ser indiretamente refletido por uma redução pós-natal significativa na excreção urinária de beta-2-microglobulina no grupo de esteroides, ao reduzir a carga filtrada dessa proteína e não ao aumentar sua reabsorção tubular, uma vez que não foram observadas diferenças significativas em sua fração de excreção. No entanto, o valor de p de 0,06 na fração de excreção de beta 2-microglobulina indica uma potencial associação que pode ser confirmada por um estudo com amostra maior ou com um período de acompanhamento mais longo, sendo essa outra limitação de nosso estudo.

O efeito dos esteroides na redução da carga filtrada de beta 2-microglobulina no néfron pode ser decorrente do efeito anti-linfoproliferativo nas células mononucleares, que são as principais fontes dessa proteína^19^. Por esse motivo, também avaliamos os fatores que afetam o aumento da beta 2-microglobulina, como infecção materna, sepse neonatal, acidose ou diminuição da filtração renal^20^, e não foram observadas diferenças significativas. A diminuição da excreção urinária de beta 2-microglobulina confirma que os esteroides não têm efeito na maturação do túbulo proximal renal no útero ou no período pós-natal, reduzindo o processo inflamatório. Nossos achados foram semelhantes aos obtidos por MacKintosh et al.^21^, que também não encontraram diferença significativa na reabsorção tubular de beta 2-microglobulina no grupo exposto à betametasona.

Em conclusão, os corticosteroides pré-natais não demonstraram diferença estatisticamente significativa na maturação dos túbulos renais em recém-nascidos prematuros, apesar dos resultados positivos obtidos em estudos com animais.
